# Crosstalk between Peroxisome Proliferator-Activated Receptors and Toll-Like Receptors: A Systematic Review

**DOI:** 10.15171/apb.2019.003

**Published:** 2019-02-21

**Authors:** Nasim Dana, Golnaz Vaseghi, Shaghayegh Haghjooy Javanmard

**Affiliations:** ^1^Applied Physiology Research Center, Cardiovascular Research Institute, Isfahan University of Medical Sciences, Isfahan, Iran.; ^2^Isfahan Cardiovascular Research Center, Cardiovascular Research Institute, Isfahan University of Medical sciences, Isfahan, Iran.

**Keywords:** Peroxisome proliferator-activated receptor, Toll-like receptor, Interaction, Crosstalk

## Abstract

As one of the four major families of pattern recognition receptors (PRRs), toll like receptors (TLRs) are crucial and important components of the innate immune system. Peroxisome proliferatoractivated receptors (PPARs) with three isoforms are transcription factors classified as a subfamily of nuclear receptor proteins, and are of significant regulatory activity in cellular differentiation, development, metabolism, and tumorigenesis. It is well established that PPARs agonists display anti-inflammatory effects through inhibition of the nuclear factor-kappa B (NF-κB) pathway, a key regulator of immune and inflammatory responses, in a sense that TLRs signaling pathways are mainly toward activation of NF-κB. Through a systematic review of previous studies, we aimed to address and clarify the reciprocal interaction between TLRs and PPARs in hope to find alternative therapeutic approaches for inflammatory diseases. Among the available scientific database, 31 articles were selected for this review. A comprehensive review of this database confirms the presence of a cross-talk between PPARs and TLRs, indicating that not only PPARs stimulation may affect the expression level of TLRs via several mechanisms leading to modulating TLRs activities, but also TLRs have the potential to moderate the expression of PPARs. We, therefore, conclude that, as a key regulator of the innate immune system, the interaction between PPARs and TLRs is a potential therapeutic target in disease treatment.

## Introduction

Toll-like receptors (TLRs) are a family of immune system glycoproteins with 13 members, 10 of which have been identified in human, and are responsible for several activities.^1^ As one of the four protein families of pattern recognition receptors (PRRs) superfamily, TLRs are responsible for recognition of a wide variety of conserved molecular structures including lipopeptide, lipoteichoic acids, lipopolysaccharides (LPS), peptidoglycans, zymogens, mannan, flagellin, viral and bacterial nucleic acids, and many other classes.^2,3^ In response to activation of TLRs through recognition of such specific compounds, a signaling process initiates by the mean of recurring adaptor proteins (with a total five members),^4^ and in most of the cases using myeloid differentiation protein 88 (MyD88) or toll/interleukin 1 receptor (TIR) domain containing adaptor inducing IFN-b (TRIF).^5^ Excluding TLR-3, the signaling pathways of all other TLRs through MyD88 are accompanied by a TIR domain-containing adaptor protein (TIRAP), which results in activation of transcription factors including nuclear factor-kappa B (NF-κB ) and activator protein-1 (AP-1).^6^ Various inflammatory stimuli can activate NF-κB in different diseases and induce transcription of proinflammatory cytokines, chemokines, adhesion molecules and matrix metalloproteinases (MMPs).^7^

Another class of transcription factors involved in modulating the inflammation responses is peroxisome proliferator-activated receptors (PPARs), a subfamily of nuclear receptor proteins with three members: I) α (alpha), expressed in liver, kidney, heart, muscle, adipose tissue, and others, II) β/δ (beta/delta), expressed in many tissues but markedly in brain, adipose tissue, and skin, and III) γ (gamma) expressed in virtually all tissues.^8^ By inactivation of p65 complexes or through inducing IκBα (as the main inhibitor of NF-κB signaling pathway), PPARs can directly attenuate the expression of inflammatory responses genes. It is also shown that through a physical interference, PPARs regulate AP-1 activity. Therefore, since many of anti-inflammatory genes are regulated by NF-κB and AP-1 pathways, PPARs agonist may have potential regulatory application in a wide range of inflammatory disorders.^9^

Necela et al showed that the activation of TLR pathways may obstruct the synthesis of PPAR messenger RNA (mRNA). Imposing such interference by TLRs involve NF-κB-activation.^10^ A consequence of attenuated PPARs expression is increasing the expression level of proinflammatory cytokines such as TNF-α and initiation of an inflammatory response.^11^

These findings suggest that there is a cross-talk between PPARs and TLRs which regulates the inflammatory response in different diseases. Hence, understanding the contrary regulatory effects and the related underlying mechanisms of TLRs and PPARs on the inflammatory signaling cascade is a must, and may lead to alternative prevention and treatment strategies for the related disease. In this study, we aimed to systemically review all the published relevant articles regarding PPAR and TLR cross-talk in different diseases.

## Materials and Methods

### Search strategy

The first step was searching the available database in MEDLINE (including PubMed and EMBASE), Scopus, ISI web of science and finally Google Scholar. A search using the Google general search engine was also done in order to find gray literature. Moreover, to identify possibly missed studies, a backward and forward citations search was done by screening all references of the collected database. In cases that the full text of an article was not available or accessible an email was sent to the first or the corresponding author, and if no response was received all the non-informative abstracts were excluded. The keywords used in collecting database from the internet were, but not excluded to, terms such as peroxisome proliferator-activated receptors, PPARs, Toll like receptor, thiazolidinedione’s (TZDs), fibrate drugs, etc. Several different searching strategies were performed to ensure the highest yield in probing related articles.

### Quality assessment of relevant studies

**Figure 1:**
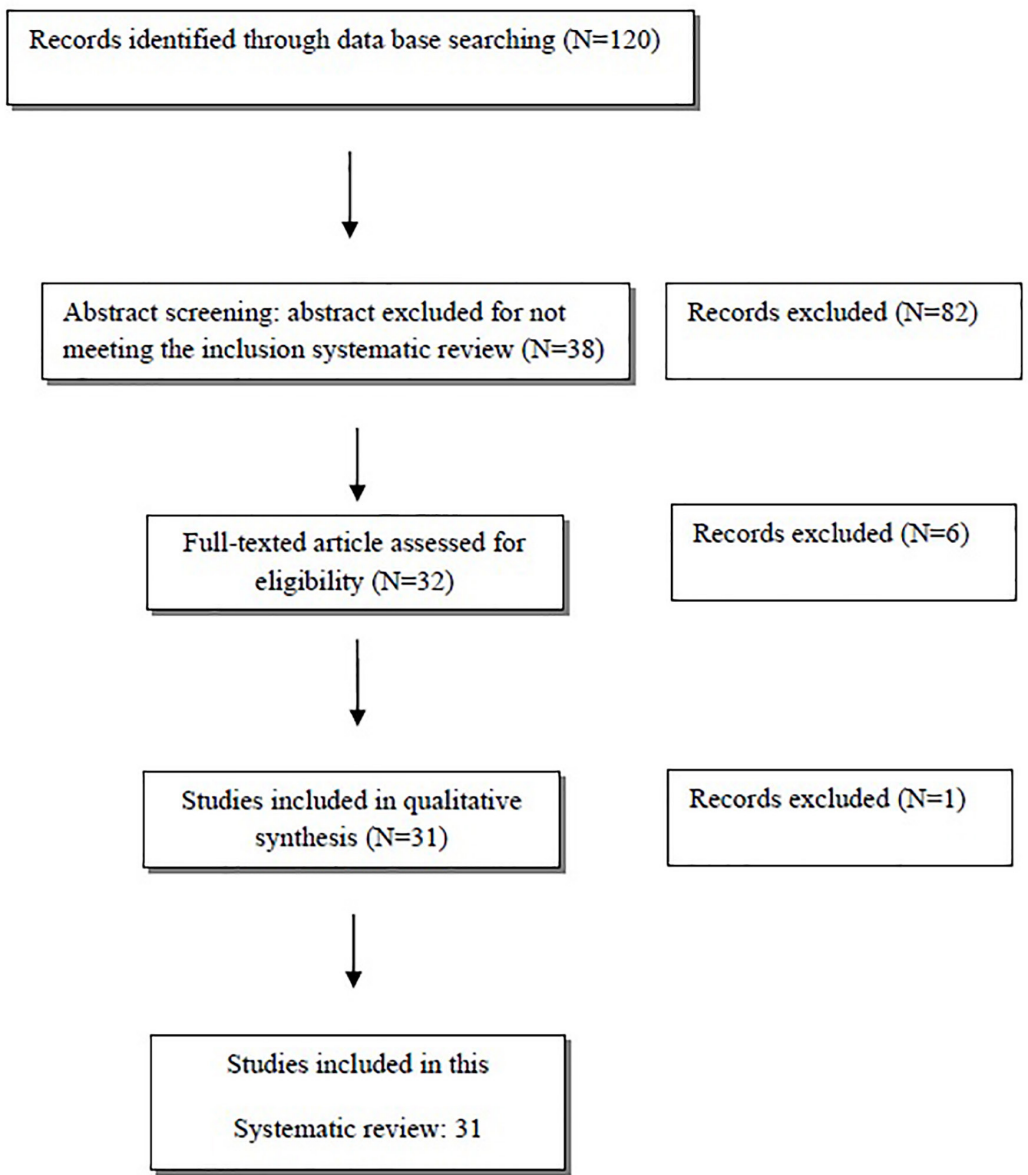
Figure 1. Literature Flow Chart.

Critical appraisal (CA) was carried out by two of the investigators. The investigators were justified and trained about the questions and meetings were held before the CA. Then, the CA has been done for 31 selected articles and in case of disagreement between investigators CA scores, it was discussed to reach an agreement, and if they did not convince, the article was CA by a third investigator. All low quality articles were excluded from the analysis/final report (Figure 1). Regardless of the language, all published articles were considered qualified if the following inclusion conditions were met: 1) English, full texts or an informative abstract in English, 2) proper study design, and 3) studies that clearly stated information about PPARs and TLRs. Our exclusion criteria were as follow: 1) studies with unclear and confusing data and 2) studies which information regarding PPARs or TLRs regulatory effects were not properly defined.

### Data extraction

The data was extracted by two of the investigators and then, rechecked by one of them. The demographic, PPARs and TLRs regulatory effects information were extracted and organized for each PPARs and TLR in Excel spreadsheets (Microsoft Office Excel, version 2007).

## Results

### TLRs and PPARs in immune response and inflammation

Zhang and Young reported that PPARs and their ligands have modulatory effects on dendritic cells and lymphocytes.^12^ Later, Appel et al studied the effects of 15-deoxy-delta12,14-prostaglandin J2 (15d-PGJ2, a PPAR-gamma natural ligand), and synthetic troglitazone (TGZ, another PPARs agonist) on the immunogenicity of human monocyte derivative from DCs upon subjecting the cells to a TLR ligands. They reported that treatment with TGZ inhibited TLR-induced DC activation through inhibiting of NF-κB and mitogen-activated protein (MAP) kinase pathways, two important regulation mechanisms of TLR and PPAR γ– mediated signaling in DCs.^13^

Macrophages are another important cell involved in the immunity. Necela et al reported that LPS as an agonist of TLR-4 receptor attenuated PPARγ expression and as a result the function of macrophages. They also showed that further pretreatment of macrophages with rosiglitazone, an antidiabetic drug and also an agonist of PPARγ, did not prevent LPS-mediated downregulation of PPARγ.^10^ They also showed that exposure to LPS moderated PPARγ mRNA synthesis in macrophages did not change the PPARγ expression level in TLR-4-knockout mice macrophages but attenuated the PPARγ expression in functional TLR-4-reconstructed cells. However, it is shown that in LPS-stimulated macrophages, NEMO, IκB, and NF-κB inhibitors suppressed the LPS-mediated downregulation of PPARγ.TLR-4 activation inhibited the PPARγ mRNA synthesis through an NF-κB-dependent mechanism. This study described a regulatory feedback loop in which in unstimulated macrophages PPARγ inhibits NF-κB-mediated inflammatory signaling, while in LPS-stimulated macrophages, when the TLR-4 was activated, the PPARγ expression was attenuated by NF-κB actions and as a result, any further potential antiinflammatory effects of PPARγ was halted.^10^

Interferon-α and –β are essential agents of antiviral innate immunity.^14^ Zhao et al studied the effects of troglitazone on IFN-β production in peritoneal primary macrophages and in response to TLR agonist (LPS and polyinosinic-polycytidylic acid [Poly (I: C)]). They showed that troglitazone repressed LPS and Poly (I: C)-induced IFN-β transcription and secretion. Troglitazone has a negative effect on the binding of IRF3 with IFN-β promoter and can inhibit LPS and Poly(I:C)-induced STAT1 phosphorylation and subsequent ISRE activation. Zhao et alshowed that in TLR-3 and TLR-4 -stimulated macrophages, PPARγ attenuated the IFN-β production by interfering with the binding of IRF3 to IFN-β promoter.^15^

In another study, it is shown that in a murine model of sepsis, using pioglitazone (another PPAR- γ agonist) resulted in an increased microbial clearance, a higher deployment of neutrophils to the infection site, and attenuated the proinflammatory cytokine production, and the increased level of IL-10. These effects were associated with a decrease in STAT-1-dependent expression of MyD88 *in vivo* and *in vitro*. Blocking the IL-10 receptor can eliminate PPARγ–mediated inhibition of MyD88 expression.^16^

Another compound that can affect PPARγ and/or TLR-4 is hypaphorine (Hy), an indole alkaloid isolated from a leguminous tree *Erythrina velutina*, which can promote sleep effects in normal mice and exhibits anti-inflammatory properties.^17^ Sun et al reported that Hy potential anti-inflammatory features might be via the regulation of TLR-4 and PPARγ I through PI3K/Akt/mTOR signaling pathways. In LPS-challenged endothelial cells, the PPARγ downregulation and TLR-4 upregulation were suppressed after treatment with Hy. They also showed that PI3K (phosphoinositide 3-kinase) and mTOR (mammalian target of rapamycin) regulated the stimulatory effect of LPS on TLR-4 expression and attenuated the PPARγ measures.^18^

Infection of macrophages with *Mycobacterium bovis* bacillus Calmette-Gue´rin (BCG) resulted in lipid accumulation and formation of lipid droplets, and also leads to mycobacterial lipid-activation of PPARγ. The mechanism of this effect is investigated by Almeida et al. Through a highly regulated mechanism, mycobacterial infection leads to PPARγ expression and later lipid metabolism and inflammation in BCG-infected macrophages which are adjusted by PPARγ activity in a TLR-2-dependent signaling pathway.^19^

In another related study by Tezera et al it is shown that in cultured Detroit cells with *Neisseria lactamica* (Nlac), through PPARγ activation and of NF-κB inhibition, the *Neisseria* itself can suppress the TLR-1/2 mediated pathogen-induced inflammation in the nasopharyngeal mucosa.^20^

Moreover, Dasu et al indicate that exposure of human monocytes and db/db mice to Pam3CSK4 (Pam; a TLR-2 ligand) and purified LPS resulted in expression of TLR-2 and -4, however, this effect was inhibited when they further administrated pioglitazone.^21^

Furthermore, Ogawa et al described that TLR-3, -4 and -9 -dependent initiation of transcriptional responses can be hampered in macrophages.^22^ It is shown that glucocorticoid receptor (GR) can hamper a large set of functionally related inflammatory response genes by disrupting p65/interferon regulatory factor (IRF) complexes. This complex is essential for TLR-4 or TLR-9 -dependent transcriptional activation, but it is not necessary for TLR-3-dependent pathways. This is through MyD88 dependent signaling and allows the GR to differentially moderate the pathogen-specific gene expression pattern.

Through a p65/IRF3-independent mechanism, both PPARγ and LXRs(liver X receptors) can prevent the overlapped transcription of some genes, and assist the GR to synergistically trans-repress a particular subsets of TLR-responsive genes.^22^

Moreover, GR, PPARγ, and LXR can inhibit proinflammatory gene expression^23^ and are capable of preventing TLR-2-induced expression of TNF-α, IL-6, and IL-8 in both monocyte-derived macrophages and monocyte-derived dendritic cells. They also can inhibit TLR-induced receptor gamma inflammatory gene expression. Timothy et al showed that PAM3CSK4, a TLR-2 ligand, can induce virus transcription in macrophages, and reported that nuclear receptors signaling can inhibit both basal and TLR-induced HIV-1 transcription.^24^

Antonopoulou et al examined the inflammatory responses in a fish, gilt-head seabream (*Sparus aurata*), and how it changed its metabolic homeostasis. In general, regarding the habitat of the fishes, they are always in danger of infection by microbes, in particular bacterial pathogens. In response to these pathogens, these creatures activate their innate immune responses such as pro-inflammatory compounds, which may also affect the non-immune tissues. Antonopoulou et al showed that the inflammation brought about by exposure to LPS was regulated the triglyceride plasma production and attenuated the amount of PPARα, β, and γ mRNAs in the hosts’ liver, while increased PPARγ production only in the anterior intestine and adipose tissues. Moreover, LPS-triggered inflammation led to a higher mRNA expression and protein activity of MAPK proteins in the fish liver. The mammalian version of these proteins is responsible for regulation of the transcription and activity of PPARs.^25^

### TLRs and PPAR in vascular smooth muscle cells

TLR-4 signaling is not only important in starting the inflammation responses, but also plays a role in pathogenesis development of atherosclerosis. Agonists of PPARα demonstrated a preventive activity in atherosclerosis probably as a result of their impact on vascular inflammation, plaque instability and thrombosis. For example, it is shown that in vascular smooth muscle cells, fenofibrate as an agonist of PPARα showed anti-inflammatory activity in which it can regulate the inflammation response triggered by angiotensin II through disturbing the TLR-4 related signaling pathways (TLR-4/IP-10/PKC/NF-κB), a hindrance against atherosclerosis.^26^

Moreover, Ji et al reported that rosiglitazone, an agonist of PPARγ, attenuated production of pro-inflammatory mediators (TLR-4, MMP-9 and TNF-α) triggered by Ang II, while on the other hand a higher level of anti-inflammatory mediators such as PPARγ and 6-keto-PGF1a were produced, in both *in vivo* and *in vitro* conditions. Moreover, by hindering TLR-4 signaling pathways (TLR-4/IP-10/PKC/NF-κB) in vascular smooth muscle cells (VSMCs) through subjecting the cells to either of small-interfering RNA (siRNA) or antagonists of TLR-4, interferon-gamma-inducible protein 10 (IP-10) siRNA, and special protein kinase C (PKC) inhibitor, they proved that the regulatory effects of rosiglitazone on Ang-II modulated inflammatory and pro-inflammatory responses are dependent on TLR-4.^27^

In another study by Ji et al, they showed that rosiglitazone attenuated the LPS-induced inflammation in VSMCs, in which this compound interfered with the activity of TLR-4 and its related domains involved in the downstream signaling, including Toll-interleukin-1 (IL-1) receptor domain containing adaptor inducing interferon-b, IRF3, and IP-10.^28^ Wu et al reported that subjecting VSMCs to OxyHb (oxyhemoglobin) resulted in an increment in the level of TLR-4 and TNF-α and caused inflammation responses. On the one hand, exposure of these cells to rosiglitazone activated the PPARγ which later attenuated cytokine release and TLR-4 expression. On the other hand, further treatment with GW9662, a specific antagonist of PPARγ, reversed the anti-inflammatory effects of rosiglitazone.^29^ Overall, these observations suggest that PPAR agonists can attenuate inflammatory response in VSMCs by interfering with TLR signaling pathways, in particular TLR-4/IP-10/PKC/ NF-κB.

### TLRs and PPAR in neuroinflammation and CNS

Microglia and astrocytes as two important cells in CNS system produce many TLRs which are important defensive agents against pathogens targeting this system; however uncontrolled expression and activity of these molecules may bring about harmful consequences for the CNS itself. Gurley et al studied the effects of different PPARγ agonists on the regulation of proinflammatory responses in primary microglia and astrocytes in presence of several TLR ligands in CNS infectious diseases. They suggest that PPARγ agonists can regulate the activities of glial cells. For example, both 15d-PGJ2 and pioglitazone attenuated the TLR ligand-induced production of IL-12 p40 by glia. However, depending on the type of TLR ligand, exposure to pioglitazone, troglitazone and 15d-PGJ2 resulted in either an increase or decrease/halt in the level of CXCL2 production.^30^ In contrast to the above study, another study suggested that in some disease TLR agonists not only are inflammation inducers but also can regulate PPARs activity. For example, PPARα and –γ of the astrocytes have an important role in inflammatory brain pathologies, another important CNS disease. Chistyakov et al reported that lipopolysaccharide, peptidoglycan, and flagellin, three bacterial antigens that act as agonists of TLR-4, TLR1/2, and TLR-5, respectively, demonstrated a time- and NF-κB -dependent inhibition activity against gene expression, protein translation and the activity of PPARα and PPARγ. In normal condition, the PPARα and PPARγ mRNAs of the naïve astrocytes have a 30 and 75 minutes’ half-life, respectively, however by induction of the TLRs their half-life increased to a nearly two-folds. On the other hand, inhibition of P38 can remove the TLR-induced stabilization on these mRNAs in a sense that the expression of these mRNAs, translation to protein and their DNA-binding activity were regulated using JNKs (c- Jun N-terminal Kinase) and p38 inhibitors. In the meantime, the transcription/translation of their isotypes (PPARα and PPARγ isotypes) was induced through the deployment of alternative regulatory mechanisms. Therefore, PPARα and PPARγ moderation in astrocytes happen at both mRNA, mRNA stability, protein and DNA-binding stages during TLR mediated responses.^31^

During endotoxin (LPS) resistance state the targeted cells have a lower expression of inflammatory proteins such as IL-6 and TNF-α, but a higher expression of anti-inflammatory genes such as IL-10 and TGFβ. Astakhova et al investigated the changes in the expression level of Cox2 in astrocytes during endotoxin tolerance state and studied the effects of PPAR agonist on the regulation of this process. It can be up- or down- regulates depending on which PPAR is activated. Under endotoxin tolerance condition, by subjecting the cells to rosiglitazone (a PPARγ agonist) there was a rise in the expression level of Cox2, while on the other hand by using GW7647 (a PPARα agonist) and L_165041(a PPARβ agonist) the Cox2 mRNA expression levels was attenuated. Therefore, PPAR agonists are potential alternative regulatory agents for Cox2 when the brain tissues are in an endotoxin-related multiple proinflammatory stimulation.^32^

Neuropathic pain is a serious harm to the nerve system. There is evidence that activities of the central neuroimmune system and inflammation of neurons play an important role in the formation and maintenance of neuropathic pain. After damage to the nerve system, microglias are activated in the spinal cord and pro-inflammatory cytokines such as TNF-α and IL-1β are secreted. Recent findings suggest that CNS microglia cells can express TLR-4, thus, inhibiting TLR-4 may reduce inflammation and alleviate the pain after nerve damage. Jia et al examined how pioglitazone affects spinal cord production of TLR-4 and cytokines, and as results, the neuropathic pain in an animal model suffering from chronic constriction injury. Their observations suggest that pioglitazone imposed a considerable suppressive effect on neuroimmune activation, in other words, it attenuated glial activation, TLR-4 and cytokines expression/production. They also showed that simultaneous use of GW9662, a PPARγ antagonist, with pioglitazone suppressed its effects.^33^

Moreover, Wu et al examined rosiglitazone effects on basilar arteries in an animal suffering from subarachnoid hemorrhage (SAH) and on vasospasm, in which they reported that by interfering with TLR-4 signaling it attenuated SAH-induced inflammatory responses.^34^

In another study, Darehgazani et al aimed at reducing inflammation by enhancing the production of PPARγ in the recombinant HEK cells carrying MD2 and TLR-4 genes. They observed that after a LPS-triggered inflammation there was an increment in the level of TNF-α and iNOS. Overexpression of PPARγ in recombinant cells disrupted LPS-triggered inflammation by interference with TLR-4/NF-κB signaling, indicating anti-inflammatory activity for PPARγ as a result of its regulatory effects on TLR-4.^35^

Immunoglobulin A nephropathy (IgAN) is another neurological disease that TLR-4 has an important role in, in which by TLR-4-related induction of proinflammatory cytokines it further results in the development of IgAN. With regard to the suppressive effects of PPARγ on TLR-4 signaling, Zou et al investigated the effects of PPARγ activation on the TLR-4-dependent progression of IgAN, *in vitro* and *in vivo*. They showed that administration of pioglitazone regulated the TLR-4-related inflammation in IgAN.^36^

Previous study suggested three major independent regulation pathways/targets for expression and activity of PPAR β/δ and its dependent genes: I) TLR/ NF-κB; II) p38, ERK/MEK, JNK MAPKs; and III) a labile protein that increases the mRNA turnover.^37^

PPAR β/δ is another potential regulatory target for inflammatory neurological diseases. There is a similarity between LPS-triggered PPAR β/δ expression and that of the Cox2, a proinflammatory compound. For example, the expression of both genes is NF-κB and p38 -dependent and is triggered after suppressing protein synthesis. After halting protein synthesis, the PPARβ/δ production is up-regulated, suggesting that a labile protein play a role in the regulation process. In contrast to Cox2, the cycloheximide- sensitive PPARβ/δ expression was not NF-κB –dependent.^37^

Chistyakov et al showed that PPARβ/δ mRNA regulation is post-transcriptionally. They measured the stability of this mRNA in TLR-stimulated astrocytes by exposing the cells to the LPS (TLR-4 agonist), peptidoglycan (TLR1/2 agonist) and flagellin (TLR-5 agonist), and reported an increment in the production and activity of PPARβ/δ. For example, not only the half-life of mRNA in LPS-exposed cells was measured at 50 minutes, but also these cells produced a higher amount of PPARβ/δ mRNA. They also showed that the production/activity of PPARβ/δ can be regulated in a similar approach to that of proinflammatory genes of TLR-agonist -stimulated astrocytes.^37^

Thus, overall, these observations strongly confirm the role of PPAR agonists in the regulation of immune responses during the CNS infection through interference with the TLR signaling pathways as a mean to alleviate the pain and reduce the damages and adverse effects imposed on the tissues of CNS.

### TLRs and PPAR in cancer

Demonstrated by Sato et al, TLR receptors can be produced by both immune and cancer cells. These molecules act against immune system activity in order to effectively facilitate cell survival and migration of the tumor during cancer development.^38^ Later it is proved that inflammation has an important role in carcinogenesis.^39^

Using the NF-κB pathway, cancer cells convert the inflammation signals into signals that lead to tumor progression, thus inhibiting NF-κB is a potential target for cancer treatment. For example, PPAR agonists are one of the inhibitory factors of the NF-κB pathway which can be used in the containment of various types of cancer. Despite the clear evidence for PPAR anti-cancer activity, the underlying mechanism has yet to be determined in many cases.^40^ With regard to the inverse effects of TLR and PPAR on NF-κB signaling, a potential underlying mechanism to explain the anti-cancer activity of PPAR is its interference with the TLRs and TLR-related activities. Wang et al studied the use of PPARγ agonist on monocytic leukemia progression. They reported that in the U937 cell line, which was subjected to LPS, rosiglitazone attenuated the TNF-α production by inhibiting TLR-4 expression, and further regulated the NF-κB signaling.^41^

A recent study by Wu et al demonstrates that rosiglitazone can also hinder the MAPK and TLR-4 pathways in esophageal cancer cells. They reported that administrating PPARγ antagonist or specific RNA interference can interrupt the rosiglitazone effects. MAPK has a TLR-4- inducible pathway, therefore it is highly possible that interfering with the TLR-4 and as a result MAPK pathways can effectively prevent the proliferation and also trigger the apoptosis of esophageal cancer cells.^42^

As demonstrated before there is limited information on the relationship between TLRs and PPAR in cancers and more research is required to well-define the interaction between the two.

### TLRs and PPARs in colon

Colon is another location where PPARγ is highly expressed and have an anti-inflammatory role. Among the nine TLRs (TLR1-9) expressed in normal epithelial cells of the colon, three (TLR-2-4) are increased in most colorectal cancer cell lines.^43^ The activation of PPARγ prevents the production of inflammatory cytokines such as TNF-α and IL-1β in the intestinal mucosa of the colon. This activity is via inhibition of NF-κB and MAP kinase signaling pathways. Eun et al showed that compared to LPS-exposed cell, stimulating HT29 cells with LPS (TLR ligand) and 15d-PGJ2 (PPARγ ligand) simultaneously, attenuated Cox2, mRNA expression, interleukin-8 protein synthesis, TLR-4 mRNA and protein levels, as well as PPARγ mRNA expression level and a decrease in the PPARγ mRNA expression. PPARγ can delay the separation of IκBα from NF-κB (which occurs as a result of LPS-stimulation), which is another confirmation for the potential application of PPARγ ligands to inhibit inflammation in intestinal epithelial cells.^44^

Moreover, Dubuquoy et al showed that the mouse commensal flora or human luminal flora can increase the expression level of PPARγ in mouse colon epithelial cells. They suggested that there is interference between PPARγ and TLR-4 signaling pathways in a sense that PPARγ can significantly prevent colitis by reducing NF-κB activity. Moderation of PPARγ production in colonic epithelial cells in response to microbial flora and/or TLR-4 activity can help in understanding the pathophysiology of chronic inflammation in IBD patients.^45^

Furthermore, despite the lack of appropriate human-base study, animal-based studies suggest a potential key role for TLRs in colon carcinogenesis. Pimentel-Nunes et al reported that in adenomas and carcinomas, of the measures of COX2 and TLR were increased, while TOLLIP(Toll interacting protein) expression was attenuated. In the case of carcinomas, the level of TLR-2 was increased while PPARγ was decreased. Continuous expression of the TLR which was accompanied by an attenuated level of TLR inhibitors led to the production of a high amount of TLR protein in lesions of colon cancer. This and bacterial-stimulation of such molecules may play a crucial role in colon carcinogenesis and tumor progression.^46^

It is well established that TLRs activities are important in IBD pathogenesis and progression, not only through their regulation but also it is possible that their dysregulation might be of importance as well. In both active and inactive ulcerative colitis and Crohn’s disease, the amount of PPARγ and TOLLIP were attenuated at both transcription and translation levels. On the other hand, stimulation of Caco-2 cell line (a colonic epithelial cell) with either the TLR ligands or the commensal flora resulted in an increment in the expression level of PPARγ and A20 (inhibitor of NF-κB ), suggesting that IBD pathogenesis and development might be associated with distinctive measurable changes in TLR-4 and TLR inhibitory proteins.^47^

In DSS-induced (dextran sodium sulphate) colitis mouse model, treatment with PEA (palmitoylethanolamide) resulted in a lower amount of pro-inflammatory compounds and ameliorate all macroscopic signs of ulcerative colitis. The anti-inflammatory role of PEA is a result of selective targeting of the S100B/TLR-4 axis on enteric glial cells, which further suppressed the NF-κB -dependent inflammation. On the one hand, in enteric glial cells PEA attenuated S100B and TLR-4 production while it does not affect other TLRs. On the other hand, using PPARα agonists (but not PPARγ) suppressed PEA activity in both mice and human studies.^48^

### TLRs and PPARs in asthma

Asthma is a common chronic inflammatory disease of the respiratory tracts. Glucocorticoids (GCs) are one of the widely used effective drugs in asthma treatment which suppress the inflammation. The epithelium of the respiratory tracts plays a key role in initiating and regulation of inflammatory response. The role of GC in the asthma management is well understood, nevertheless, less is known about its effects and underlying mechanism on the epithelium of asthmatics. Diez et alused a network analysis to examine the potential role of PPAR and interferon-1 in GCs activity against asthmatic inflammation. They showed that TLR and PPAR signaling pathways are involved in inflammation. With regard to the GCs mechanism of action, it is reported that TLR-dependent interferon production is a key target. Administration of GC resulted in upregulation of the PPAR signaling pathway which leads to attenuating/suppressing the TLR-dependent interferon production.^49^

## Conclusion

In conclusion, the progressively increasing studies about TLRs activity will result in a better and more detailed understanding of the importance of these molecules for human health as well as the underlying mechanism involved in either the pathogenesis of some disease and/or the treatment process. Upregulation of certain TLRs is important for the development of innate response such as inflammation, production of cytokines, etc, whereas, some others are important in the regulation of inflammatory response to prevent excessive inflammation. An important mechanism to regulate the action of TLRs involves the PPARs. Interestingly, there is enough evidence demonstrating a cross-talk between TLRs and PPARs, in other words, not only PPARs can alter TLRs activity the opposite is valid as well, and TLRs can alter PPARs effects (Table 1). For example, using PPARs agonist or antagonist has been practiced in many diseases, in which the activator or blocker ligands changed the TLRs activity by activation or deactivation of certain PPARs signaling pathways, and vice versa. Thus, in conclusion, there is a regulatory feedback loop between PPARs and TLRs signaling pathways.

**Table 1:** Cross talk between PPAR and TLR agonists in different cells and tissues

**Authors/Year**	**Ligand and mode of interaction**	**Tissue or cell**	**Main conclusions**
Almeida et al 2009	PPARgamma antagonist GW9662	Immunity	PPARγ acts in a TLR2-dependent signaling pathway as a key molecule of lipid metabolism and inflammation in BCG-infected macrophages. Treatment of HMEC-1 with pioglitazone suppressed LPS-induced TLR-4 expression.
Appel et al 2005	15d-PGJ2 troglitazone	Dendritic cells	Treatment of TLR-stimulated dendritic cells with troglitazone, suppresses the TLR effects through inhibiting NF-κB and MAP kinase pathways.
Astakhova et al 2015	PPAR agonists GW7647, L-165041, and rosiglitazone	Astrocytes	The expression level of COX2 mRNA, in cells that are at an endotoxin-resisting state, is regulated by PPARs agonists. GW7647 and L-165041 attenuate its production while rosiglitazone induces higher expression.
Chistyakov et al 2015	lipopolysaccharide, peptidoglycan, and flagellin, which are agonists of TLR4, TLR1/2, and TLR5, respectively	Astrocytes	LPS, flagellin and peptidoglycan, three agonists of TLRs, are time and NF-κB –dependent PPARs inhibitors in astrocytes.
Chistyakov et al 2014	agonists of TLR4, TLR1/2, and TLR5, using lipopolysaccharide (LPS), peptidoglycan, and flagellin, respectively	Astrocytes	The stimulation of TLR receptors on the cell surface of astrocytes increased PPARβ/δ level at both transcription and translation levels. Moreover, exposure of the cells to LPS resulted in higher expression level of PPARβ/δ mRNA.
Dasu et al 2009	Pioglitazone	Monocytes	Treatment of db/db mice with pioglitazone attenuated TLR4 and TLR2 level, and as a result, MyD-88 and Nf-kB levels in peritoneal macrophages.
Diez et al 2012			Glucocorticosteroid receptors suppressed TLR-dependent interferon production by upregulation of PPAR signaling pathway.
Dubuquoy et al 2003	GED, pioglitazone, 5-ASA, GW9662, poly I:C	Colon	There might be Interference between the PPARγ and TLR4 signaling pathways epithelial cells of the colon. The microflora can lead to an increment in the expression of PPARγ in epithelial cells, and as a result regulated Nf-kB -dependent inflammatory responses‏.
Esposito et al 2014	PPARα antagonist MK866; PPARγ antagonist GW9662	Colon	The PEA anti-inflammatory effects are through targeting the S100B/TLR4, which inhibits Nf-kB -dependent inflammatory responses, and only PPARα (but not PPARγ) antagonist eliminated the PEA effects in both mice and humans -base studies.
Eun et al 2006	LPS 15d-PGJ2	Colon	The inflammation of intestinal epithelial cells was prevented by PPARγ agonists. ‏
Fernandes et al 2016		Colon	TLR4 is the only TLR which was significantly produced in IBD disease, while the expression level of both PPARγ and TOLLIP was downregulated at both transcription and translation levels. This demonstrate a potential TLRs dependent pathogenesis in the case of IBD.
Ferreira et al 2014	pioglitazone	Immunity	In the mouse model of sepsis, the treatment with pioglitazone attenuated the production of pro-inflammatory cytokines such as TNF-α and IL-1b while elevated IL-10 levels. This effect of pioglitazone was associated with a decreased expression of MyD88 in *in vitro* and *in vitro*. Blocking IL-10 receptor suppresses this effect.
Gurley et al 2008	15d-PGJ(2)	Microglia and Astrocyte	Pioglitazone and troglitazone demonstrated opposing effects on microglial CCL2 production that were TLR ligand-dependent. 15d-PGJ2 and pioglitazone attenuated TLR-dependent production of IL-12 p40.
Hanley et al 2011	TLR2 ligand PAM3CSK4	Macrophages	TLR2 signaling pathways is responsible for a higher HIV-1 presentation on the cell surface of macrophages and dendritic cells. It also increases the expression and production of pre-inflammatory cytokines which can be attenuated through a PPARγ-dependent signaling pathways, demonstrating possible role of such mechanism in the pathogenesis of HIV-1.
Ji et al 2009	Fenofibrate	Vascular smooth muscle cells	The inhibitory effect of fenofibrate on the inflammatory responses of VSMCs induced by angiotensin II, is TLR4-dependent. Fenofibrate interferes with downstream signaling pathways, such as protein kinase C, IP-10 and NF-kB, and reduces the inflammation in VSMCs.
Ji et al 2011	Rosiglitazone decreasing‏ TLR4	Vascular smooth muscle cells	Rosiglitazone interference with TLR4 and its signaling pathways including Toll-interleukin-1, TRIF, IRF3, and IP-10, attenuated the inflammatory responses.
Ji et al 2009	Rosiglitazone	Vascular smooth muscle cells	Rosiglitazone is a potential treatment for atherosclerosis, in which it regulates angiotensin-mediated inflammatory responses in VSMCs. The targeted signaling pathways by rosiglitazone are ERK1/2/TLR4/IP-10/PKC/NF-kB.
Jia et al 2016	PPARγ agonist, pioglitazone PPAR-γ antagonist GW9662		Pioglitazone suppressed the neuroimmune activation characterized by glial activation, the production of cytokines and expression levels of TLR-4. Utilizing GW9662, the antagonist of PPARγ, disrupted the anti-inflammatory effects of pioglitazone.
Necela et al 2008	LPS	Macrophage	In macrophages, there is a regulatory feedback loop connecting PPAR and TLR signaling pathways, in which PPAR inhibits NF-κB-dependent inflammation in unstimulated macrophages, while on the other hand, 20TLR4 stimulation, and attenuated PPAR expression through a NF-KB-dependent mechanism.
Ogawa et al 2005	LPS	Macrophage	Simultaneous activation of GR and PPARγ or GR and LXRs resulted in additive/synergistic inhibition of subsets of TLR4 responses in vivo and in vitro.
Pimentel-Nunes et al 2012	-	Colon	In general, in carcinomas a higher level of TLR2 and a lower PPAR-γ is expressed. There was a trend towards decreased TOLLIP and PPAR-γ from normal mucosa to adenoma/carcinoma. These suggest a possible role of this signaling pathways in development and progression of colon cancers.
Sun et al 2017	LPS	HMEC-1 cells	Suppressing the TLR4 or using an activator (agonist) of PPAR-γ such as pioglitazone obviously attenuated the amount of pro-inflammatory cytokines in LPS-treated HMEC-1 cells.
Tezera et al 2011	LPS, synthetic triacyl lipopetide Pam3Cys-Ser-(Lys) 4 (Pam3Cys, 1 µg/mL) and lipoteichoic acid PPAR-g antagonist T0070907	Nasopharyngeal epithelial cells	*Neisseria lactamica* is capable of PPAR-γ induction in nasopharyngeal mucosa, and as a result, suppresses the NF-κβ activity. Therefore, by employing such mechanism it attenuated the TLR ½ -dependent pathogen-induced inflammatory responses.
Wang et al 2011	Rosiglitazone	Inflammatory and tumor-derived U937 cells	Treatment of by rosiglitazone suppressed TLR4 expression and attenuated TNF-α production, by regulating NF-kB signaling pathway.
Wu et al 2016	rosiglitazone	EC109 and TE10 esophageal cancer cells	Rosiglitazone activation of PPARγ suppressed proliferation and induced apoptosis of esophageal cancer cells by inhibiting TLR4-dependent MAPK pathway.
Wu et al 2011	Rosiglitazone	Brain	PPARγ agonist such as rosiglitazone can attenuate the SAH-induced inflammatory responses by interfering with TLR4 signaling.
Wu et al 2010	Rosiglitazone antagonist for PPARgamma, GW9662	Vascular smooth muscle	PPARγ agonist such as rosiglitazone attenuated TLR4 expression and as a result production of the related cytokines. But, using PPARγ antagonist suppressed such effects. The anti-inflammatory effects suggest a potential therapeutic approach for treatment of vasospasm following SAH.
Zhao et al 2011	Troglitazone LPS and poly(I:C)	Macrophage	PPAR-γ negatively regulates IFN-β production in TLR3- and 4-stimulated macrophages by preventing IRF3 binding to the IFN-β promoter.
Ghaedi et al 2016	LPS Transient expression of EGFP-PPARγ	HEK cell line	PPARγ can prevent LPS-triggered inflammation through regulation of TLR4 signaling pathways.
Antonopoulou et al 2017	LPS	Immunity	LPS-triggered in Gilthead Seabream affected the plasma level of triglyceride and is accompanied by a lower expression of PPARα, β, and γ mRNAs in the liver. It also resulted in higher expression level and activity for MAPK.
Zou et al 2017	Toll‏ like receptor 4 inhibitor (TAK242) pioglitazone	Renal	Pioglitazone can suppress the TLR4-dependent inflammatory responses, a key activity in pathogenesis of immunoglobulin A nephropathy (IgAN), in rat model.

## Ethical Issues

Not applicable.

## Conflict of Interest

None.

## Acknowledgments

This article is driven from a PhD thesis promoted at Isfahan University of Medical Sciences with a grant number of No. 394617. This work was financially supported by Iran National Science Foundation (INSF) [grant No. 95844116].
